# Evaluation of Sleep Quality Among People Living With Type 2 Diabetes Mellitus in Taif, Saudi Arabia: A Cross-Sectional Study

**DOI:** 10.7759/cureus.64124

**Published:** 2024-07-09

**Authors:** Abdulmajeed Algethami, Fawaz K Alfahmi, Muhanna A Alhusayni, Saeed A Bamusa, Yusra I Alsalmi, Alhanouf F Alboqami, Amjad F Aldosari

**Affiliations:** 1 Internal Medicine, Taif University, Taif, SAU; 2 College of Medicine, Taif University, Taif, SAU

**Keywords:** saudi arabia., risk factors, sleep quality, sleep disorders, diabetes mellitus, t2dm, type 2 diabetes mellitus

## Abstract

Background: Type 2 diabetes mellitus (T2DM) is characterized by high blood glucose levels, which are highly associated with poor sleep quality, cardiovascular disease, and pathological changes. This research examines the relationship between sleep quality and T2DM and compares it with nondiabetics within the Taif community. The findings of this study will provide valuable insights and recommendations to enhance the overall health quality in Taif, Saudi Arabia.

Methodology: A cross-sectional study was conducted on 547 patients with T2DM between December 1, 2023, and April 1, 2024, in Taif. The sleep quality was assessed using the Sleep Quality Questionnaire (SQQ). Data were collected using an online questionnaire with two parts: primary demographic data and an assessment of sleep quality using the SQQ.

Results: Our study enrolled 814 participants, including 547 with T2DM and 267 nondiabetics. Participants with T2DM had poorer sleep quality, with a median score of 21 vs. 25 (*P *< 0.001). Significant factors affecting sleep quality included gender (*P *= 0.002), marital status (*P *= 0.023), and job status (*P *= 0.023). Nondiabetics had better sleep quality (76%) than participants with T2DM (61.1%). Males, married, and employed individuals reported higher sleep quality scores.

Conclusions: Research indicates that individuals with T2DM experience lower sleep quality than the general population, particularly among female, unmarried, and unemployed individuals. To enhance sleep quality in patients with T2DM, it is essential to increase awareness, provide education on proper sleep habits, and highlight the importance of effective diabetes management, screening for sleep disorders, and consistent monitoring.

## Introduction

Diabetes mellitus (DM) is a significant public health problem characterized by metabolic irregularities in carbohydrate, lipid, and protein metabolisms that lead to hyperglycemia due to unsuitable gluconeogenesis and glycogenolysis processes. There are various classifications of DM, with the main types being type 1 DM (T1DM), which is attributed to an immune system abnormality, and type 2 DM (T2DM), which is linked to genetic abnormalities or lifestyle factors like obesity, smoking, and lack of physical activity. Additionally, gestational diabetes mellitus (GDM) is distinguished by hormonal fluctuations during pregnancy [1-3]. DM is one of the most common disorders worldwide. DM affects all age groups, including 529 million worldwide [4]. Sleep is a normal and essential process for life and brain health. Sleep is involved in metabolism, regulation of hormones, and the response of an immune system [5]. Sleep disorders are common and are highly associated with stroke and vascular brain injury. In patients with depression, there is a link between sleep disorders and suicide and T2DM [6-10]. Sleep disorders can impact various medical conditions, reducing the quality of life [5].

T2DM is a chronic metabolic disorder that endangers the health and economy of every nation, particularly developing ones. Due to insulin resistance or low amounts of secretion, insulin resistance or reduced secretion of insulin leads to elevated blood sugar levels. This may be caused by increased risk factors of T2DM, including smoking, decreased physical activity, and a high-caloric diet. T2DM is affected by some factors and can occur at any age. Still, the group most affected is adults over 65 [2,11]. In the last few years, the prevalence of T2DM has increased worldwide, particularly in developing countries. Recent studies predict that the prevalence rate of T2DM will increase from 5.9% in 2021 to 9.5% in 2050 [4]. In 2023, the estimated prevalence of T2DM in Saudi Arabia was around 16.4% [12]. Conversely, in 2013, Kuwait reported a higher prevalence rate of approximately 25.4% [13]. Furthermore, in 2009, Qatar documented a prevalence rate of approximately 17.79% [14]. T2DM is associated with various complications, which can be categorized into short-term *acute* complications such as diabetic ketoacidosis (DKA) and hyperosmolar hyperglycemic state (HHS), and long-term *chronic* complications that can be further classified as microvascular or macrovascular complications. However, it is essential to note that chronic complications are more prevalent compared to acute complications like DKA and HHS [1,2,15]. Inadequate sleep quality has been identified as a significant contributing factor to poor glycemic control [1,16]. The significance lies in the correlation between poor glycemic control and the development of complications associated with T2DM, such as cardiovascular diseases, retinopathy, neuropathy, and pathological problems [17,18].

 Many studies have tried to measure sleep quality in people with diabetes. A similar survey conducted in Makkah City, Saudi Arabia, reported a similar prevalence of poor sleep quality (63.7%) [19]. Studies conducted in various locations, including Jazan (55.4%) and Abha (72.2%) in Saudi Arabia, have reported prevalence rates of poor sleep quality among individuals with T2DM [18,20]. Furthermore, a study conducted in Amman, Jordan, outside of Saudi Arabia, revealed an even higher prevalence rate of 81% for poor sleep quality among T2DM patients [21]. Among T2DM Chinese patients, excessive sleep duration is prevalent and negatively impacts their quality of life [22]. In Taiwan, it is common for T2DM patients to experience disrupted night-time sleep and excessive daytime sleepiness, with more than 50% of patients reporting sleep disturbances in a particular study [23].

 This research examines the relationship between sleep quality and T2DM and comparison with non-diabetics within the Taif community. The findings will provide valuable insights and recommendations to enhance the overall health quality in Taif, Saudi Arabia. By examining the influence of sleep quality on T2DM, potential interventions and tactics to enhance the welfare of individuals with diabetes in the local community can be determined. The findings will aid in creating specific approaches that can adequately tackle sleep-related concerns and ultimately improve the health results of T2DM patients in Taif. 

## Materials and methods

Study design, duration, and setting

A cross-sectional survey was conducted in the endocrinology department at Taif University between December 1, 2023, and April 1, 2024.

Participants

Inclusion criteria were as follows: (1) patients diagnosed with T2DM who met the World Health Organization’s diagnostic criteria and those of the American Diabetes Association [24]; (2) nondiabetics, to contrast their findings with those diagnosed with T2DM; and (3) patients 18 years and older of either sex. Exclusion criteria were as follows: (1) patients diagnosed with T1DM, gestational diabetes, diabetes insipidus, and other types of diabetes; (2) patients diagnosed with T2DM under the age of 18.

Determination of sample size

The required sample size was predestined using Raosoft (version 3.8, Raosoft, Inc., Seattle, WA), 95% confidence interval (CI), 5% margin of error, and 50% response distribution. Past studies showed that the prevalence of T2DM in the Taif region was 20,000 [25,26]. A sample size of 377 participants was calculated. Despite this, the response rate was unexpectedly low, likely due to the high rates of diabetes among the elderly and the difficulty they encountered in completing the questionnaire. People who were eligible to participate in this study were sent the questionnaire. Finally, we obtained 547 individuals with T2DM and 267 without a history of diabetes who met the criteria.

Data collection

The questionnaire was divided into two parts. The first part included primary demographic data, and the second part included an assessment of sleep quality using the questionnaires for quality [27]. The first part contained primary demographic data on age, gender, nationality, occupation, and marital status. The second part contained the SQQ, which is a validated scale. SQQ is a nine-item response to a questionnaire that assessed the sleep quality of individuals participating in the study over the past month. SQQ is divided into the first question: How long does it take you to fall asleep? The second question: If you wake up one or more times during the night, estimate how long you are awake in total (add up all the times you are awake). The third question was: If your final wake-up time occurs before you intend to wake up, how much earlier is this (0-15 minutes, 4 points; 16-30 minutes, 3 points; 31-45 minutes 2 points; 46-60 minutes 1 point; >60 minutes 0 points)? Fourth question: How would you rate your sleep quality (Very Good, 4 points; Good, 3 points; Average, 2 points; Poor, 1 point; and Very Poor, 0 points)? Fifth question: How many nights a week do you have a problem with your sleep (0-1, 4 points, 2, 3 points; 3, 2 points; 4, 1 point; 5-7, 0 points)? The sixth question: Has it affected your productivity, concentration, or ability to stay awake? The seventh question, Has it affected your energy, relationships, or mood? The eighth question: Has it troubled you in general (Not at all, 4 points; a little, 3 points; somewhat, 2 points; much, 1 point; and very much, 0 points)? The last question: How long have you had a problem with your sleep (I do not have a problem/<month, 4 points; 1-2 months, 3 points; 3-6 months, 2 points; 7-12 months, 1 point; >1 year, 0 points)? Participants were classified into four groups according to their sleep issues. The first group had severe sleep problems and needed help. The second group experienced sleep problems and required an examination of their habits. The third group had a good night’s sleep but needed improvement. The fourth group had a good night’s sleep and proceeded with their practice. The participants were advised to continue their sleep routine based on the results (Table 1).

**Table 1:** Survey designed to assess the quality of sleep in individuals diagnosed with type 2 diabetes mellitus.

Questions	Choice A	Choice B	Choice C	Choice D	Choice E	Choice F	Choice G
Do you consent to being a participant in the research endeavor?	Yes	No	-	-	-	-	-
Gender	Male	Female	-	-	-	-	-
Age	19-21 years old	22-29 years old	30-39 years old	40-50 years old	Larger than 50 years old	-	-
Nationality	Saudi	Non-Saudi	-	-	-	-	-
Marital status	Single	Married	Divorced	Widowed	-	-	-
Education degree	Illiterate	Elementary	Middle school	High school	Bachelor	Diploma	Postgraduates (Master’s or Doctorate)
Job status	Unemployed	Student	Public Sector Officer	Private Sector Officer	-	-	-
Monthly income	<5,000 SAR	5,000-10,000 SAR	10,000-20,000 SAR	20,000-30,000 SAR	20,000-30,000 SAR	-	-
Living at home with individuals under 18 (including children and adolescents)?	Yes	No	-	-	-	-	-
Having first-class relatives (parents, brothers or sisters, children) who are health professionals?	Yes	No	-	-	-	-	-
How long does it take you to fall asleep?	0-15 min	16-30 min	31-45 min	46-60 min	more than 60 min	-	-
If you wake up one or more times during the night, estimate how long you are awake in total.	0-15 min	16-30 min	31-45 min	46-60 min	more than 60 min	-	-
If your final wake-up time occurs before you intend to wake up, how much earlier is this	I don’t wake up too early / up to 15 min. early	16-30 min	31-45 min	46-60 min	more than 60 min	-	-
How would you rate your sleep quality?	Very good	Good	Average	Poor	Very poor	-	-
How many nights a week do you have a problem with your sleep?	0-1	2	3	4	5-7	-	-
Consider the past month, to what extent has poor sleep, affected your productivity, concentration, or ability to stay awake?	Not all	Not all	Somewhat	Much	Very much	-	-
Consider the past month, to what extent has poor sleep, affected energy, relationships, or mood?	Not all	Not all	Somewhat	Much	Very much	-	-
Consider the past month, to what extent has poor sleep, troubled you in general?	Not all	Not all	Somewhat	Much	Very much	-	-
Consider the past month, to what extent has poor sleep, and how long have you had a problem with your sleep?	I don’t have a problem	1-2 months	3-6 months	7-12 months	More than one year	-	-

Statistical methods

The data were extracted and revised in an Excel sheet. Statistical analysis was conducted using the IBM SPSS computer program (version 26.0, IBM Corp., Armonk, NY). Categorical variables were described in numbers and percentages. Continuous, non-normally distributed variables were reported as the mean (standard deviation [SD]) as well as the median and interquartile range (IQR). The sleep quality score was calculated for all nine questions included. Each question had five options, and the score ranged from 0 to 4. The total score was the sum of all questions (out of 36 points). The association between total score and independent variables was conducted using the Mann-Whitney test. The Chi-square test categorized and compared the sleep quality score to different variables. *P*-values less than 0.05 were considered statistically significant.

Ethical considerations

The Taif University Scientific Research Ethics Committee reviewed and approved the study that involved human patients (HAO-02-T-105).

## Results

Our study enrolled 814 participants. Among them, 547 were patients with T2DM, with a mean (SD) age of 40.62 (15.88) years, and 267 were participants with no history of diabetes, with a mean (SD) age of 42.17 (12.10) years. The majority of respondents were Saudis (94.9% among patients with T2DM and 85.5% among participants with no diabetes history), females (63.1% among patients with T2DM and 59.3% among participants with no diabetes history), and married (61.7% among patients with T2DM and 77.3% among participants with no diabetes history). Most participants had a high school degree (86.1%), and 34.6% were jobless. Only 5.4% of the participants earned more than 30,000 SAR monthly. The majority (79.4%) lived with individuals under 18, and 43.2% had first-class relatives who were health professionals, as shown in Table 2.

**Table 2:** Demographic characteristics of the participants (N = 814). T2DM, type 2 diabetes mellitus

Participants		T2DM (*N* = 547)	No diabetes (*N* = 267)	Total (*N* = 814)
Age (Years)	Mean (SD)	40.62 (15.88)	42.17 (12.098)	41.13 (14.756)
	Median (IQR)	43 (29)	45 (15)	43 (25)
	Min-Max	18-87	18-84	18-87
Parameters	Category	*n *(%)		
Gender	Male	190 (36.9)	61 (40.7)	251 (37.7)
	Female	325 (63.1)	89 (59.3)	414 (62.3)
Nationality	Saudi	487 (94.9)	124 (85.5)	611 (92.9)
	Non-Saudi	26 (5.1)	21 (14.5)	47 (7.1)
Marital status	Single	168 (32.6)	26 (17.3)	194 (29.2)
	Married	318 (61.7)	116 (77.3)	434 (65.3)
	Divorced	13 (2.5)	5 (3.3)	18 (2.7)
	Widowed	16 (3.1)	3 (2)	19 (2.9)
Education degree	Illiterate	4 (0.7)	-	4 (0.5)
	Elementary	2 (0.4)	-	2 (0.2)
	Middle school	8 (1.5)	-	8 (1.0)
	High school	432 (79.4)	261 (97.8)	693 (86.1)
	Bachelor	80 (14.7)	-	80 (9.9)
	Diploma	12 (2.2)	-	12 (1.5)
	Postgraduates (Master’s or Doctorate)	6 (1.1)	-	6 (0.7)
Job status	Unemployed	174 (33.8)	56 (37.3)	230 (34.6)
	Student	119 (23.1)	19 (12.7)	138 (20.8)
	Public Sector Officer	183 (35.5)	62 (41.3)	245 (36.8)
	Private Sector Officer	39 (7.6)	13 (8.7)	52 (7.8)
Monthly income	<5,000 SAR	225 (43.7)	52 (34.7)	277 (41.7)
	5,000-10,000 SAR	90 (17.5)	27 (18)	117 (17.6)
	10,000-20,000 SAR	140 (27.2)	49 (32.7)	189 (28.4)
	20,000-30,000 SAR	33 (6.4)	13 (8.7)	46 (6.9)
	>30,000 SAR	27 (5.2)	9 (3.5)	36 (5.4)
Living at home with individuals under 18 (including children and adolescents)?	Yes	406 (78.8)	122 (81.3)	528 (79.4)
	No	109 (21.2)	28 (18.7)	137 (20.6)
Having first-class relatives (parents, brothers or sisters, children) who are health professionals	Yes	332 (60.7)	130 (48.7)	352 (43.2)
	No	215 (39.3)	137 (51.3)	462 (56.8)

Table 2 illustrates participants’ responses regarding sleep quality questions. For patients with T2DM, about two-thirds of them (32%) took from 16 to 30 minutes to fall asleep, and 32.5% took less than 15 minutes to be awake in total during the night. Among nondiabetic participants, 43.1% took 16 to 30 minutes to fall asleep, and 53.6% took less than 15 minutes to be awake in total during the night. 

For T2DM, most participants either did not wake up too early or up to 15 minutes early (31.6%) or between 16 to 30 minutes earlier than the intended time (34.9%). Almost one-third (32.4%) reported having one night or none of a sleeping problem. Only 7.7% rated their sleep quality as very good; meanwhile, the majority rated it as an average (43.1%). Among nondiabetic individuals, most participants either did not wake up too early or wake up 15 minutes early (28.8%) or woke up between 16 and 30 minutes earlier than the intended time (44.2%). Almost half of them (49.1%) reported having one night or none of a sleeping problem. Only 13.5% rated their sleep quality as very good.

For the past month, T2DM participants reported poor sleep affected their productivity a little (25%) to somewhat (38.6%), as well as their relationships and mood a little (25.2%) to somewhat (35.1%). Moreover, one-third (30.2%) reported that poor sleeping somewhat troubled them in general. Unfortunately, 36.2% reported having problems sleeping for more than one year. For nondiabetic participants, sleep quality did not affect their productivity at all (20.2%), as well as their relationships and mood (16.5%). Moreover, one-third (31.8%) reported that poor sleeping generally troubled them a little. More than half of them (55.4%) did not report having a sleep problem or had it for less than a month. Full details are shown in Table 3.

**Table 3:** Participants’ responses regarding sleep quality questions.

Parameters	Category	Diabetic (*N *= 547)		Nondiabetic (*N* = 267)	
		n	%	n	%
How long does it take you to fall asleep?	0-15 minutes	132	24.1	73	27.3
	16-30 minutes	175	32.0	115	43.1
	31-45 minutes	97	17.7	44	16.5
	46-60 minutes	67	12.2	14	5.2
	>60 minutes	76	13.9	21	7.9
If you wake up one or more times during the night, estimate how long you are awake in total.	0-15 minutes	178	32.5	143	53.6
	16-30 minutes	159	29.1	61	22.8
	31-45 minutes	92	16.8	29	10.9
	46-60 minutes	58	10.6	14	5.2
	More than 60 minutes	60	11.0	20	7.5
If your final wake-up time occurs before you intend to wake up, how much earlier is this	I don’t wake up too early/up to 15 minutes early	173	31.6	77	28.8
	16-30 minutes	191	34.9	118	44.2
	31-45 minutes	99	18.1	33	12.4
	46-60 minutes	40	7.3	12	4.5
	>60 minutes	44	8.0	27	10.1
How would you rate your sleep quality?	Very good	42	7.7	36	13.5
	Good	150	27.4	98	36.7
	Average	236	43.1	98	36.7
	Poor	75	13.7	25	9.4
	Very poor	44	8.0	10	3.7
How many nights a week do you have a problem with your sleep?	0-1	177	32.4	131	49.1
	2	117	21.4	57	21.3
	3	111	20.3	27	10.1
	4	50	9.1	16	6.0
	5-7	92	16.8	32	12.0
Consider the past month, to what extent has poor sleep.					
Affected your productivity, concentration, or ability to stay awake?	Not all	86	15.7	54	20.2
	A little	137	25.0	72	27.0
	Somewhat	211	38.6	100	37.5
	Much	79	14.4	31	11.6
	Very much	34	6.2	10	3.7
Affected energy, relationships, or mood?	Not all	71	13.0	44	16.5
	A little	138	25.2	77	28.8
	Somewhat	192	35.1	98	36.7
	Much	105	19.2	37	13.9
	Very much	41	7.5	11	4.1
Troubled you in general?	Not all	66	12.1	46	17.2
	A little	148	27.1	85	31.8
	Somewhat	165	30.2	64	24.0
	Much	116	21.2	57	21.3
	Very much	52	9.5	15	5.6
How long have you had a problem with your sleep?	I don’t have a problem/	192	35.1	148	55.4
	1-2 months	61	11.2	33	12.4
	3-6 months	65	11.9	16	6.0
	7-12 months	31	5.7	6	2.2
	>1 year	198	36.2	64	24.0

In addition, the total sleep quality scores among the patients with T2DM and nondiabetic participants are described in Table 4. The total score ranged from 0 to 36 out of 36, with a median (IQR) of 21 (11) among patients with T2DM and 25 (10) among nondiabetic participants. This difference in the median total sleep quality scores was statistically significant (*P *< 0.001), where the diabetic participants had lower total scores of good sleep quality than nondiabetic patients.

**Table 4:** Total scores of the participants' answers regarding sleep quality.

			*P*-value
The total score of the sleep quality among T2DM	Mean (SD)	20.938 (7.724)	<0.001
	Median (IQR)	21 (11)	
	Min-Max	0-36	
The total score of the sleep quality among no diabetic participants	Mean (SD)	23.92 (7.307)	
	Median (IQR)	25 (10)	
	Min-Max	0-36	

Moreover, the total sleep quality of the T2DM and nondiabetic participants was categorized as having great to good sleeping shape and having some to severe sleep problems, as shown in Table 5. Having a great shape of sleep was more prevalent among nondiabetic individuals (36.3%) than diabetic patients (23.6%).

**Table 5:** The scale of sleep quality among T2DM and nondiabetic participants. T2DM, type 2 diabetes mellitus

Parameters	Category	Number	Percentage
Scale of sleep quality among T2DM	Your sleep problems seem to be severe	38	6.9
	You have some sleep problems	175	32.0
	Your sleep is in good shape	205	37.5
	Your sleep is in great shape	129	23.6
Scale of sleep quality among nondiabetics	Your sleep problems seem to be severe	9	3.4
	You have some sleep problems	55	20.6
	Your sleep is in good shape	106	39.7
	Your sleep is in great shape	97	36.3

By categorizing sleep quality into good or poor quality, a significant association was seen between the presence of DM and sleep quality (*P *< 0.001). The nondiabetic participants had better sleep quality (76%) than the participants with T2DM (61.1%), as shown in Table 6.

**Table 6:** Association between the presence of diabetes mellitus and sleep quality.

Factor		Sleep quality, *n* (%)		*P*-value
		Good	Poor	
Presence of DM	Nondiabetic	203 (76)	64 (24)	<0.001
	T2DM	334 (61.1)	213 (38.9)	

In Table 7, the significant factors affecting sleep quality were illustrated. Gender (*P *= 0.002), marital status (*P* = 0.023), and job status (*P* = 0.023) had an impact on overall sleep quality scores. Males had higher sleep quality scores than females, with a median of 23 vs. 20, respectively. Married participants had higher sleep quality scores than unmarried participants, with a median of 22 vs. 20, respectively. Additionally, employed participants had higher sleep quality scores than unemployed participants, with a median of 22 vs. 20.

**Table 7:** Impact of the individuals’ characteristics and sleep quality scores among patients with T2DM. T2DM, type 2 diabetes mellitus

Factors		Median (IQR)	*P*-value
Age	≤40 years	21 (10.75)	0.568
	>40 years	21 (12)	
Gender	Male	23 (11)	0.002
	Female	20 (11)	
Nationality	Saudi	21 (11)	0.456
	Non-Saudi	20 (15.5)	
Marital status	Married	22 (12)	0.023
	Unmarried	20 (11)	
Educational level	High school or less	21 (11)	0.993
	Bachelor's or postgraduate degree	21 (12)	
Job-status	Unemployed	20 (12)	0.023
	Employed	22 (11)	
Income	≤10,000 SAR	21 (11)	0.78
	>10,000 SAR	21 (12)	
Living at home with individuals under the age of 18 (including children and adolescents)	Yes	21 (11)	0.957
	No	20 (12)	
Their first-class relatives (parents, brothers or sisters, children) are health professionals	Yes	21 (11)	0.737
	No	21 (11)	

## Discussion

Poor sleep quality is a significant issue for patients with DM as it increases the risk of insulin resistance and DM-related complications. Effective sleep management can improve diabetes and enhance sleep quality [28]. Research has proven that insufficient sleep duration increases the risk of developing T2DM [29]. We conducted this study to investigate the impact of DM diagnosis on sleep quality in Taif City, Saudi Arabia.

Our study demonstrated that individuals with T2DM had significantly lower sleep quality scores than nondiabetic patients. Similarly, studies have shown that individuals with diabetes experience more sleep disturbances compared to those without diabetes [30-32]. Another study showed that diabetic individuals had reported a significantly higher prevalence of sleep disturbance in Qatar compared to the general population [33]. Also, research has revealed that while the prevalence of sleep disorders in the general population is around 20%, it is nearly doubled among people with T2DM, which aligns with our results [34-36].

Concerning the rate of poor sleep quality in Saudi Arabia, our research findings indicate that a significant percentage of patients with T2DM in Taif City, Saudi Arabia (61.1%) experience poor sleep quality. A similar study conducted in Makkah City, Saudi Arabia, reported a similar prevalence of poor sleep quality (63.7%) [19]. Similar results were obtained from studies conducted in Jazan (55.4%) and Abha (72%) in Saudi Arabia [18,20]. In areas outside Saudi Arabia, another study conducted in Amman, Jordan, showed an even higher prevalence of poor sleep quality among patients with T2DM (81%) [21]. Additionally, poor sleep is prevalent among T2DM Chinese patients, which is inversely associated with life quality [22]. In Taiwan, patients with T2DM commonly experience poor nighttime sleep and excessive daytime sleepiness; more than 50% of patients in one study reported sleep disturbances [23].

Regarding the factors affecting sleep quality in patients with T2DM, our study showed poorer sleep quality among females, unmarried, and unemployed participants. In line with our findings, females had a significantly higher risk of experiencing poor sleep quality [21]. Other studies reported that female patients were more likely to have poor sleep quality [21,22,32,37,38]. In addition, unemployed and unmarried participants have a significantly higher risk of poor sleep quality [21], consistent with our findings. The reason for our findings could be that unemployment can lead to psychological distress, including anxiety, low self-esteem, and depression. These can result in poor sleep quality and quantity [39,40]. For marital status, a previous study showed that marriage can lead to better sleep quality and that co-sleeping with a partner is associated with longer sleep [41]. Furthermore, prior research has indicated that disruptions in romantic relationships, such as separation, divorce, or widowhood, can result in an increased likelihood of experiencing sleep disturbances [42].

Limitations

The nature of the study design limits the validity of our findings. It is a cross-sectional observational study using a self-administered questionnaire conducted in a single place in Saudi that may lead to collecting data at a single point in time. In addition, it is essential to note that this study may have considered only some potential factors that could impact sleep quality. As a result, it is highly recommended that future studies be conducted as generalized studies and a more comprehensive investigation of all possible variables that could affect sleep quality among T2DM. It is essential to focus on weight measurement, particularly concerning body mass index (BMI). Additionally, it is crucial to consider the patient's medical history and measure the duration of diabetes by referencing data from multiple medical centers.

## Conclusions

The study revealed poor sleep quality levels among patients with T2DM than the general population. Being female, unmarried, and unemployed participants decreased sleep quality scores among diabetic participants. Therefore, raising awareness about the impact of diabetes on sleep and educating patients about good sleep practices are crucial for improving sleep quality among patients with T2DM. Additionally, it emphasizes the importance of effective diabetes management, including medication adherence, exercise, and a healthy diet. Screening for sleep disorders and regular monitoring and follow-up appointments could also help improve sleep quality.
